# Ectopic Pregnancy Observed With Kyleena Intrauterine Device Use: A Case Report

**DOI:** 10.7759/cureus.35637

**Published:** 2023-03-01

**Authors:** Samantha R Singer, Julian Melchor, Sarah J Ripps, Jennifer Burgess

**Affiliations:** 1 Academic Institution, Florida State University College of Medicine, Tallahassee, USA; 2 Obstetrics and Gynecology, Women’s Health Specialist OBGYN, Stuart, USA

**Keywords:** kyleena, risk factors, levonorgestrel, intrauterine device, iud, ectopic pregnancy

## Abstract

Kyleena (levonorgestrel 19.5 mg), a type of intrauterine device (IUD), has an efficacy rate of 99% in preventing pregnancy. Because the overall failure rate of IUDs is low, ectopic pregnancy (EP) with IUD use is uncommon. This case reports an EP observed in a female with the Kyleena IUD in place. She had no known risk factors for an EP, which makes this case noteworthy. Ultrasound and surgery confirmed a 4 cm EP in the ampulla of the left fallopian tube. Insufficient evidence exists to conclude whether the Kyleena IUD has a higher risk of EP compared to other hormonal IUDs. As the Kyleena IUD becomes a more popular option for women in search of an effective contraceptive, patients and clinicians should be aware of this potential risk. Our case emphasizes that continued research on the prevalence of EP with Kyleena use is necessary.

## Introduction

Intrauterine devices (IUDs) are low-maintenance and long-acting contraceptive options for women. The risk of pregnancy in a study that observed all brands of IUDs was 1% [1]. Kyleena (levonorgestrel 19.5 mg) is one of the newest IUDs available and has an efficacy rate of 99% in preventing pregnancy for each year of use, and 98.6% efficacy over five years. Research has shown that if an IUD fails, the device is likely to prevent implantation within the uterus, leaving the embryo to implant in an extrauterine location [2].

An ectopic pregnancy (EP) is an implantation of the embryo or gestational sac in an extrauterine site, most commonly in the fallopian tube. While some EPs can be asymptomatic, the most common presentation is a woman of reproductive age with first-trimester bleeding and abdominal pain [3]. The severity and location of the abdominal pain can vary, ranging from colicky abdominal to lower pelvic pain [4]. If the EP is unruptured, it can present with localized abdominal pain. On the other hand, a ruptured EP can cause generalized abdominal pain due to hemoperitoneum and may lead to life-threatening hemorrhage [3,4]. Other symptoms include normal pregnancy discomforts (e.g., breast tenderness, urinary frequency), syncope, vomiting, diarrhea, rectal pressure, dyschezia, and many others [3,4].

Diagnosis should be clinically suspected when a patient presents with symptoms of an EP as well as cervical motion tenderness, adnexal tenderness, or hemodynamic instability on the physical examination [4]. Confirming the diagnosis involves a transvaginal ultrasound, a serum beta-human chorionic gonadotropin (β-hCG) test, or visualization during surgery [3]. The transvaginal ultrasound allows the clinician to visualize the gestational sac or embryo in an extrauterine site [4]. The rate of increase in serum β-hCG levels can aid in the diagnosis of an EP as well [4]. A slower-than-expected rise in β-hCG can signify either a pregnancy loss or an EP, thus serial β-hCG levels should be measured over 48 hours [4]. Other differential diagnoses to consider are spontaneous abortion, subchorionic hematoma, gestational trophoblastic disease, physiological bleeding, and cervical, vaginal, or uterine pathology [3].

Treatment for an EP depends on whether the patient is hemodynamically stable or unstable. If the patient is hemodynamically unstable, immediate surgical management is recommended [3]. If the patient is hemodynamically stable, then treatment may range from medical management (e.g., intramuscular methotrexate) to surgical management (e.g., salpingostomy) depending on the clinical severity and patient compliance [4]. Patient compliance is crucial to monitor serial β-hCG levels which must reach less than 15 IU/L for conclusive resolution [5].

This article was previously presented as a poster presentation at the 2022 University of Central Florida (UCF) Global Health Conference on January 15th, 2022.

## Case presentation

**Figure 1:**
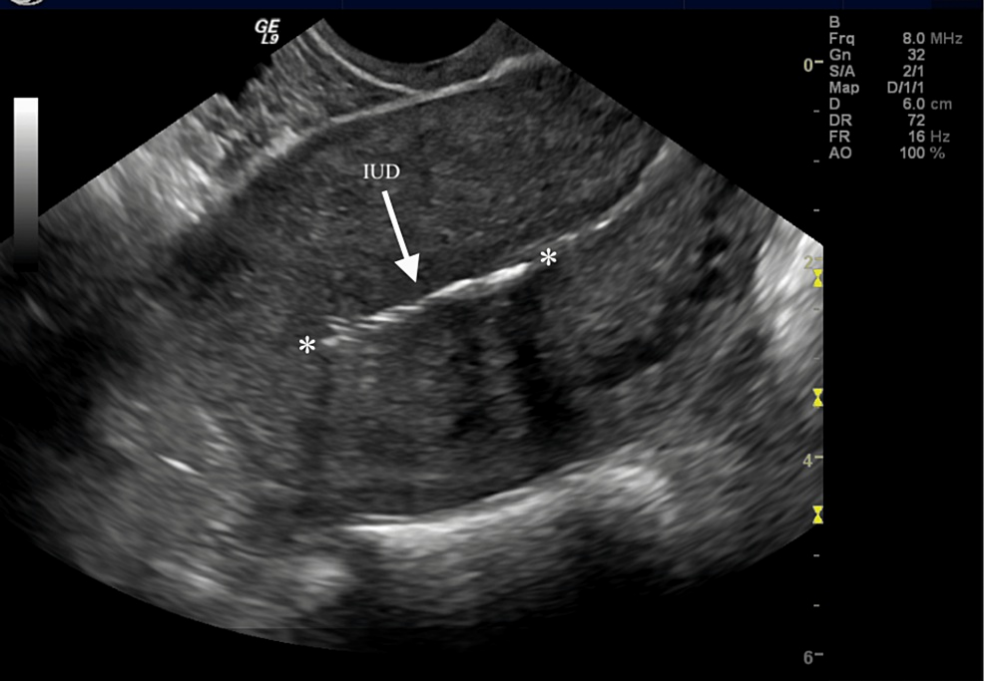
Ultrasound of the IUD in proper position within the uterus at the time of initial placement. IUD = intrauterine device

**Figure 2:**
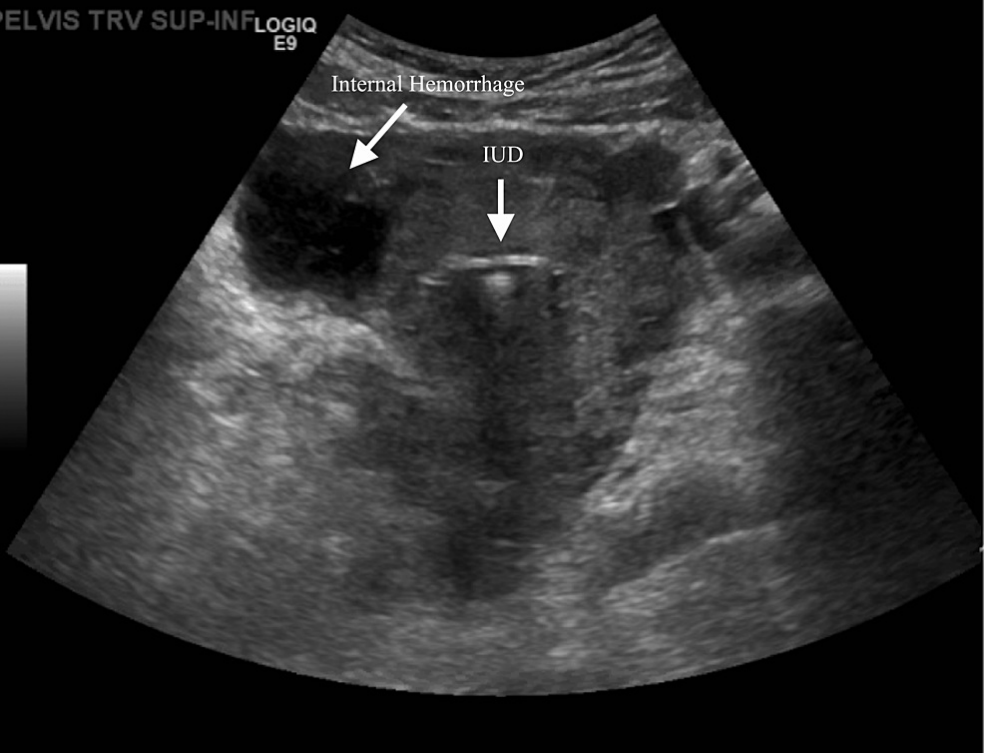
Ultrasound of the IUD in proper position with evidence of internal hemorrhage. IUD = intrauterine device

Our case centers on a young female who had a Kyleena IUD placed in September 2018, two months after giving vaginal birth. She had an IUD placed in the proper position with ultrasound imaging (Figure 1). Her menstrual cycle on the IUD consisted of intermittent bleeding that was lighter than normal menses. She had no history of pelvic inflammatory disease, sexually transmitted infections, or any significant medical history, family history, or social history that would have increased her risk of an EP. Three years later at 31 years of age, she was found to have a positive pregnancy test with a β-hCG of 5,108 and vaginal bleeding while on Kyleena. Ultrasound results discovered the presence of free fluid in the abdominal cavity highly indicative of an EP (Figure 2).

**Figure 3:**
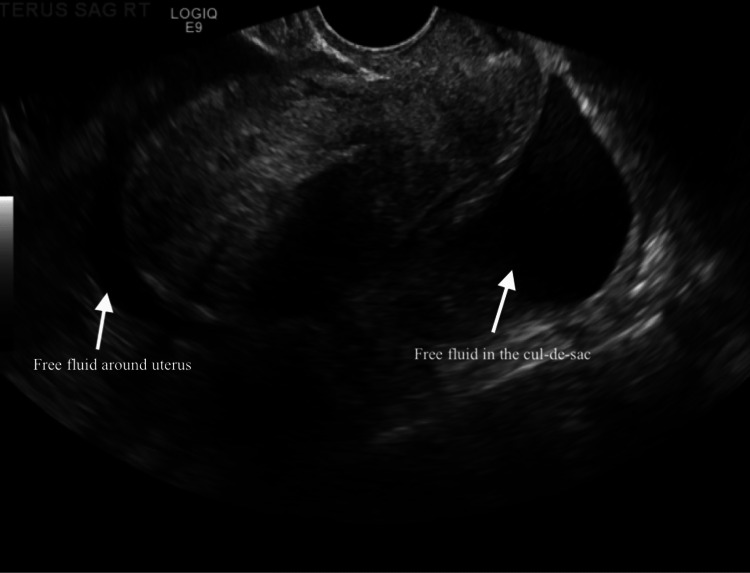
Ultrasound showing hemorrhaging around the uterus and cul-de-sac.

**Figure 4:**
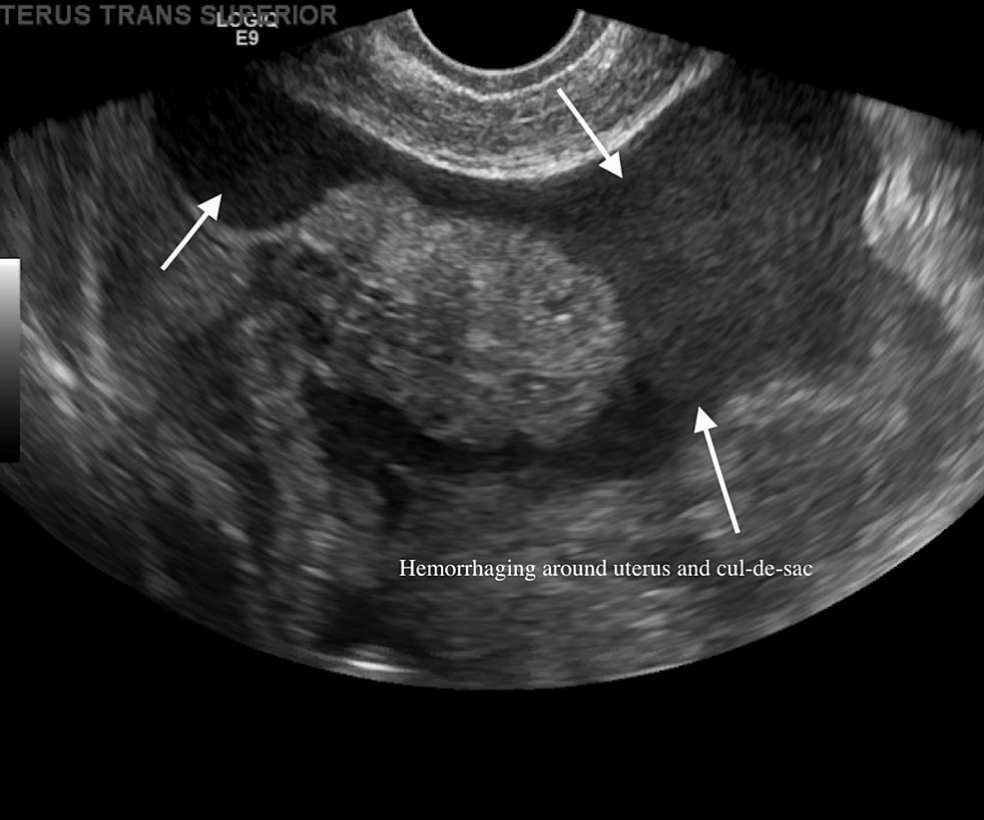
Alternate ultrasound view of hemorrhaging around the uterus and cul-de-sac.

Real-time sonographic imaging of the pelvic content was evaluated from both transabdominal and endovaginal approaches. The uterus was anteverted and the bladder was empty. The uterus measured 7.5 × 6.2 × 5.0 cm. The endometrial thickness was irregular, measuring up to 7 mm. This irregularity was explained by her intermittent menstrual pattern after three years on the Kyleena IUD. Both ovaries were normal in appearance with small follicular cysts. Free pelvic fluid was seen on both sides of the pelvis and scattered throughout the abdomen (Figures 3, 4). This was a first-trimester ruptured EP with hemoperitoneum and acute blood loss leading to anemia (hemoglobin 9.9 g/dL). Surgery confirmed a 4 cm EP in the ampulla of the left fallopian tube.

## Discussion

The risk of EP due to IUD use is rare [1,6]. However, there are certain factors that increase a patient’s risk including pelvic inflammatory disease, sexually transmitted infection, endometriosis, migration of the levonorgestrel-releasing intrauterine system, age, scarring, previous pregnancies, appendectomy, prior fallopian tube surgery, and many others [7]. Notably, our patient’s past medical history did not include any of these risk factors, emphasizing the uniqueness of this case. Historically, most patients with an EP have a significant past medical history leading to the diagnosis, while this patient did not.

There are several types of IUDs, with the most common forms including copper and levonorgestrel [8]. Copper IUDs do not secrete any hormones, but create an inhospitable environment within the uterus due to the release of copper ions [9]. Levonorgestrel IUDs, including Kyleena, Mirena, Skyla/Jaydess, all differ in size, the amount of hormone secreted, and the approved duration of use. Specifically, Kyleena is one of the smallest and secretes the least amount of hormone. Due to the minimal hormonal secretion, small size, and long duration of use, Kyleena is becoming one of the more appealing options for patients interested in an IUD [6].

Our research on the topic of hormonal IUDs and EPs emphasizes the rare nature of this case as data show pregnancies occur less than 1% of the time with IUD contraceptive use [6]. This case brings to light the potential risk of EP associated with Kyleena, even without any typical EP risk factors. As Kyleena has become a more commonly used option for hormonal IUDs, patients and clinical providers should be aware of this risk [10]. Future research using randomized control trials should be conducted to better assess the risk of ectopic and intrauterine pregnancies with Kyleena compared to other hormonal IUDs.

## Conclusions

A paucity of research on this topic makes it difficult to conclusively state that Kyleena causes a higher risk of EPs. However, this case provides evidence supporting the possibility that EPs with Kyleena may be less rare than previously believed to be. Continuing to monitor future cases long-term as well as conducting further research is crucial to determine if there is a greater association of EP with Kyleena IUD use.
